# CHARCTERIZATION OF FEEDING OF CHILDREN UNDER 24 MONTHS IN UNITS CARED BY THE FAMILY HEALTH STRATEGY

**DOI:** 10.1590/1984-0462/2020/38/2019027

**Published:** 2020-03-16

**Authors:** Joelânia Pires de Oliveira Souza, Camila Silva Ferreira, Danielle Mendonça Buiatti Lamounier, Leandro Alves Pereira, Ana Elisa Madalena Rinaldi

**Affiliations:** a Universidade Federal de Uberlândia, Uberlândia, MG, Brazil.

**Keywords:** Family Health Strategy, Complementary food, Survival analysis, Breast feeding, Estratégia Saúde da Família, Alimentação complementar, Análise de sobrevida, Aleitamento materno

## Abstract

**Objective:**

To describe the median age of introduction and regular food intake by young children in units of the Family Health Strategy.

**Methods:**

Cross-sectional study with 283 young children chosen by uniform stratified cluster sampling. Socio-demographic data on the mother and the child’s food intake were collected (age of food introduction and weekly frequency). Foods were grouped into *in natura*/minimally processed (G1); culinary ingredients (G2); processed (G3) and ultra-processed (G4). The survival analysis was applied to define the median age of food introduction and the chi-square test was used to compare the frequency of food intake, according to the age range (0-5.9; 6-11.9; and 12-23.9 months old).

**Results:**

The median duration of exclusive breastfeeding and breastfeeding were three and 19 months, respectively. The age of median food introduction of G1 was six months, except for eggs, milk and coffee (12 months). For oil and salt (G2), the median was 6 months, and for sugar (G2), seven months. The median age of introduction of most food of G4 was 12 months; for infant formulas, it was seven months; cookies and baby food, eight months. Most food had not yet been introduced for children under six months old. For children from six to 11.9 months old, the regular consumption of G4 (≥5 days/week) was higher for cookies (23.8%), bread (21.2%), infant formulas (21.2%) and baby food (35%); and for children from 12 to 23.9 months old, it was higher for cookies (31.2%), bread (57.5%) and baby food (48.7%).

**Conclusions:**

Food introduced and consumed on a regular basis was mainly *in natura*, at all ages. Processed and ultra-processed food presented a higher frequency of consumption after 12 months old.

## RESUMO

**Objetivo:**

Descrever a idade mediana de introdução e consumo regular de alimentos por crianças menores de 24 meses em unidades da Estratégia Saúde da Família.

**Métodos:**

Estudo transversal realizado com 283 crianças selecionadas por amostragem por conglomerado estratificada uniforme. Foram coletados dados sociodemográficos da mãe e do consumo alimentar da criança (idade de introdução e frequência semanal). Os alimentos foram agrupados em: *in natura*/minimamente processados (G1); ingredientes culinários (G2); processados (G3) e ultraprocessados (G4). Utilizou-se análise de sobrevida para definição da idade mediana de introdução dos alimentos e teste do qui-quadrado para comparação da frequência de consumo segundo a faixa etária (0 a 5,9; 6 a 11,9; e 12 a 23,9 meses).

**Resultados:**

A duração mediana do aleitamento materno exclusivo foi de três meses e do aleitamento materno, de 19 meses. A mediana de introdução dos alimentos do G1 foi de seis meses, exceto para ovo, leite e café (12 meses). Para óleo e sal (G2), a mediana foi de 6 meses e para o açúcar (G2), de sete meses. A mediana da maioria dos alimentos do G4 foi de 12 meses, para fórmulas foi de sete meses, biscoitos e alimentos infantis, oito meses. A maioria dos alimentos ainda não tinha sido introduzida para crianças menores de seis meses. Para crianças de seis a 11,9 meses, o consumo regular (≥5 dias/semana) de G4 foi superior para biscoitos (23,8%), pão (21,2%), fórmulas (21,2%) e alimentos infantis (35,0%); e para crianças de 12 a 23,9 meses foi para biscoitos (31,2%), pão (57,5%) e alimentos infantis (48,7%).

**Conclusões:**

Os alimentos introduzidos e consumidos regularmente foram principalmente alimentos *in natura*, em todas as idades. Alimentos processados e ultraprocessados apresentaram maior frequência de consumo após os 12 meses.

## INTRODUCTION

Breastfeeding (BF) and timely complementary feeding are one of the most promising interventions to prevent child mortality. It is estimated that from 13% and 6% of all deaths of children under five could be avoided with breastfeeding and complementary healthy feeding, respectively.^1^ Expanding BF to an almost universal level could prevent 823 thousand deaths per year for children under five years old. ^2^ The 1,000-day period was considered a window of opportunities for all health interventions, mainly due to the intense brain and tissue growth, the development of the immune system and human capital, besides creating and developing healthy eating habits.^3^ ^,^ ^4^

Recent studies on the dietary pattern of Brazilian children under 24 months indicate similar characteristics regarding the early introduction of liquids in the first month of life,^4^ ^,^ ^5^ early introduction of ultra-processed food, such as soft drinks, sweetened beverages, crackers, snacks and dairy beverages, and reduced consumption of vegetables and fruits.^5^ ^,^ ^6^ ^,^ ^7^ ^,^ ^8^ ^,^ ^9^ ^,^ ^10^ ^,^ ^11^ The introduction of all types of food basically occurs up to 12 months, and the guidelines for introducing food with higher salt, sugar and fat content is only after 24 months old.^11^ The early introduction of sweetened beverages was seen in 32% of Brazilian children under two years old, which is associated with the regular consumption of sweetened beverages by the parents, at home, and the habit of watching TV for more than three hours a day.^12^

Given scarce national-based research on the topic, the present study is added to the regional studies already carried out to build the scenario of infant and young child feeding in Brazil. Seen that, the objectives of this study were describing the median age of food introduction and its consumption for young child , registered in units of the Family Health Strategy (FHS) in Uberlândia City, Minas Gerais State.

## METHOD

Cross-sectional study with 283 children under two years old, registered in 34 units of the FHS in Uberlândia City, Minas Gerais State. Data collection was performed from January to November 2012, after approval by the Research Ethics Committee of Universidade Federal de Uberlândia (Protocol CEP/UFU No. 213/11).

According to data from the Brazilian Primary Care Information System (SIAB), there were 3,910 children under two years old registered in 42 units of the FHS in Uberlândia City, in 2012. The sample calculation was made considering the following data: reference population of 3,910 children, a 95% significance level, a margin of error of 5% for sampling and 50% for response variables (food eaten by children, classified as “yes” or “no”). After adopting these parameters, the sample number totaled 350 children under two years old, whose data were collected in the units of the FHS. From the completion of the pilot study, the absence of children on scheduled days was frequent, which showed to be difficult to reach the planned sample for each unit. Thus, the sample was obtained by uniform stratified cluster, including eight children in each stratum (this figure resulted from dividing 350 children by 42 units). Considering the number of children in each unit was different, we applied the sampling weight, which corresponds to the inverse of the probability an individual has for being selected according to the sample design. This weighting factor makes the children selected in each unit represent all registered children and approximates a simple random sample, minimizing the bias of one cluster sample.

The final sample consisted of 283 children (80.8% of the initially calculated sample, and 5.6% of sample error) registered in 34 units of the FHS. Data collection was not performed in eight units of the FHS, two of which were located in a rural area of difficult access, four were not operating during the data collection period, one shared the physical facility with another team, and the other one was the place of the pilot study. The children who participated in the study pilot were not included in the sample, because the objective was to test the research instrument, estimate the time for applying the questionnaire, assess if the order of questions was pertinent, and verify if the responsible for the children understood the questions. A total of 12 children under 24 months old was investigated in the pilot study (4.1% of all children interviewed). Analysis was performed with and without the inclusion of children from the pilot study (283+12=295) and there was no change in the results.

Data collection was performed in the waiting rooms of the units of the FHS by three undergraduate Nutrition students, previously trained to apply the questionnaires. Data collected were children’s age and gender, maternal age and education, family economic classification according to the Brazilian Criteria of Economic Classification,^13^ child enrollment in nurseries/early childhood schools (yes/no), mother’s participation in educational groups of breastfeeding and complementary feeding during pregnancy (yes/no), infant feeding guidance during prenatal consultations (yes/no), and data on feeding.

We analyzed the age of food introduction (retrospective information) and the frequency of food consumption of the week prior to the interview, when food had already been introduced. These two aspects were assessed because the age of food introduction represents how early food is offered and the frequency of food consumption, how important it is to expose children to recommended food and to avoid other types of food in this age group.

The food list of the frequency questionnaire was elaborated based on the study by Monteiro et al. ^14^ for four different reasons: interest in analyzing the consumption of processed and ultra-processed food in this age group; reduced number of food consumption markers present in the Brazilian Food and Nutrition Surveillance System (SISVAN) for children under two years old; the non-existence of a food frequency questionnaire, proposed in the Food Guide for children under two; and the non-existence of the list of processed and ultra-processed food in official documents by the World Health Organization (WHO).^15^ The categories of answers were: did not introduce the food until the interview moment; did not consumed it over the last week; consumed it in 1 day/week; consumed it from two to four days/week, and from five to seven days/week. Subsequently, the variables for food consumption were defined as non-regular (<5 days/week) and regular (≥5 days/week).^16^

Foods were organized into four groups according to the nature, extent and purpose of processing: G1 - *in natura/*minimally processed food; G2 - culinary ingredients; G3 - processed food; G4 - ultra-processed food.^17^ We chose to add “greengrocers” (*quitandas*) to the food frequency questionnaire, a regional term from Minas Gerais State that refers to cakes, cheese crackers, corn bread (*broas*), biscuit and other sweet or salty preparations that are traditionally served with coffee.

Descriptive analyzes of socioeconomic data and the frequency of food consumption were expressed according to three age groups (0‒5.9; 6‒11.9; and 12‒23.9 months old), due to different dietary recommendations. The comparison between these categorical variables was performed with the chi-square test. Numerical quantitative variables were expressed as medians with the 1st and 3rd quartiles (Q1 and Q3, respectively), for non-adherence to the normal distribution (Shapiro-Wilk test). The median time of food introduction was estimated with the Kaplan-Meier estimator, because it refers to a variable that involves time and, mainly, because it contains incomplete information for children whose mothers have not yet introduced food (censorship detected). Food introduction and censorship were considered a failure when the mother had not yet introduced the food. A significance level of 5% was adopted. Statistical analysis was performed using Stata 12^®^ software.

## RESULTS

The study was conducted with 283 children with a median age of seven months (Q1; Q3: 4.12 months old). Regarding maternal education, 42% of mothers had completed high school (≥11 years) and 46% were classified in the economy class D (lower class). More than 95% of mothers received prenatal care; 78% reported receiving breastfeeding and infant feeding guidelines during consultations, and 49% participated in groups on the topic (Table 1). The percentage of children enrolled in nurseries/early childhood education was 6.7%, of which 1.7% were under 6 months old, 11.1% were from 6 to 12 months old, and 10% were over 12 months old.

**Figure 1:**
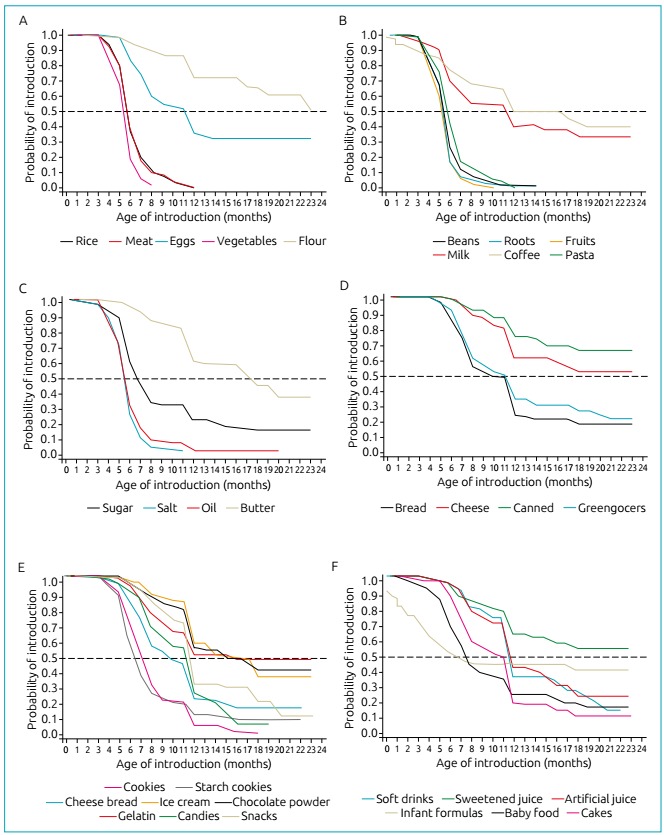
Probability of introduction of *in natura* and minimally processed food (A and B), culinary ingredients (C), processed food (D) and ultra-processed food (E and F) by infants registered in basic family health units. Uberlândia City, Minas Gerais State, 2012.

The percentage of children who were exclusively breastfed (EBF) among children under six months was 24.9%, and 72.3% were breastfed, with a reduction of these percentages as they became older (Table 1). The median duration of EBF was three months and that of BF was 19 months. In the *in natura* food group (G1), the median introduction for rice, beans, meat, vegetables, roots, fruits and pasta was six months; and for egg, milk and coffee, 12 months. By the eighth month, vegetable introduction was reported to 100% of children; until the tenth month for fruit and the twelfth month for meat, rice and pasta. For culinary ingredients (G2), the median introduction was six months for oil and salt; and seven months for sugar. As for processed foods (G3), the median introduction for bread and greengrocers was ten months, for flour cookies, seven months, and cheese had a slightly later probability of introduction, with a median of 18 months (Figure 1).

**Table 1:** Sociodemographic data and healthcare of children registered in basic family health units according to the age group. Uberlândia City, Minas Gerais State, 2012.

	Age group (months)			**p-value**
	<6 (n=122)	6 to 11.9 (n=81)	12 to 23.9 (n=80)	
Sociodemographic				
Gender (child)				
Male	46.2 (36.9-55.7)	61.8 (49.6-72.6)	57.0 (44.9-68.4)	0.136
Female	53.8 (44.3-63.1)	38.2 (27.3-50.4)	43.0 (31.6-55.1)	
Maternal education				
<8 years	15.4 (8.7-22.1)	11.0 (3.3-18.7)	16.2 (8.0-24.5)	0.616
8 to 10 years	40.4 (30.9-49.9)	36.5 (25.1-47.9)	35.9 (24.3-47.4)	
≥11 years	44.2 (34.6-53.7)	52.5 (40.7-64.3)	47.9 (35.8-60.1)	
Economic class				
B (B1+B2)	---	2.0 (1.0-5.1)	---	0.167
C (C1+C2)	51.0 (41.4-60.6)	56,5 (44.9-67.9)	54.6 (42.4-66.8)	
D+E	48.9 (39.4-58.6)	41,5 (30.1-52.9)	45.4 (33.2-57.6)	
Maternal age	25.1 (21.6-28.8)	24.7 (18.2-35.4)	24.8 (22.0-29.3)	0.445
Health care		% (95%CI)		
Prenatal visits (number)				
<6	2.9 (0.9-8.9)	6.8 (2.9-15.4)	9.5 (4.5-19.0)	0.068
≥6	97.1 (91.4-99.1)	93.2 (84.6-97.1)	90.5 (81.0-95.5)	
Participation in educational groups (breastfeeding)				
No	17.1 (11.3-25.2)	20.4 (12.5-31.7)	25.0 (16.5-36.2)	0.174
Yes	82.9 (74.8-88.7)	79.6 (68.3-87.5)	75.0 (63.8-83.5)	
Breastfeeding children (%)*				
No	17.2 (11.0-26.0)^a^	17.2 (10.1-27.6)^a^	55.2 (43.1-66.8)^b^	<0.001
Yes	82.8 (74.0-89.0)	82.8 (72.4-89.9)	44.8 (33.2-56.9)	

Data is expressed in % (95% Confidence Interval) or in median (1st-3rd quartle); *different letters: p-value<0.05. The difference is significant between the category <6 and 12 to 23.9 months and between 6 to 11.9 and 12 to 23.9 months (different letters).

Roughly all food in the ultra-processed group (G4) had a median introduction up to 12 months, except for ice cream (median=14 months) and chocolate powder (median=15 months). Ready milk and infant formulas had a median of seven months; and cookies and children’s food, eight months. Food, such as gelatin, chocolate, candies, snacks, soda, artificial juice and pasta had a median introduction of 12 months (Figure 1).

The introduction of food from all groups and culinary ingredients (oil, sugar and salt) did not occur for most kids under six months old (Tables 2, 3 and 4). For 100% of children in this age group, the introduction of ice cream, chocolate powder and soda was not reported.

**Table 2:** Food consumption of *in natura* or minimally processed food (G1) by infants registered in basic family health units. Uberlândia City, Minas Gerais State, 2012.

Food	**Age group (months)**			p-value
	<6	6 to 11.9	12 to 23.9	
	%			
Rice				
Not introduced	91.8	21.3	0.0	<0.001
<5 times	6.2	21.2	5.0	
≥5 times	2.0	57.5	95.0	
Beans				
Not introduced	86.6	10.0	1.3	<0.001
<5 times	6.2	16.3	6.2	
≥5 times	7.2	73.7	92.5	
Meat				
Not introduced	90.7	15.0	0.0	<0.001
<5 times	5.2	22.5	12.5	
≥5 times	4.1	62.5	87.5	
Eggs				
Not introduced	98.9	65.0	32.5	<0.001
<5 times	1.0	33.8	66.3	
≥5 times	0.0	1.2	1.2	
Vegetables				
Not introduced	79.4	7.5	0.0	<0.001
<5 times	7.2	6.3	6.3	
≥5 times	13.4	86.2	93.7	
Roots				
Not introduced	81.4	7.5	1.3	<0.001
<5 times	6.2	41.3	60.0	
≥5 times	12.4	51.2	38.7	
Fruits				
Not introduced	71.1	6.3	0.0	<0.001
<5 times	13.4	13.7	8.8	
≥5 times	15.5	80.0	91.2	
Milk				
Not introduced	92.8	60.0	36.3	<0.001
<5 times	1.0	7.5	3.7	
≥5 times	6.2	32.5	60.0	
Coffee/tea				
Not introduced	89.7	63.8	46.3	0.001
<5 times	9.3	30.0	42.5	
≥5 times	1.0	6.2	11.2	
Pasta				
Not introduced	97.7	18.8	0.0	<0.001
<5 times	7.2	61.2	91.3	
≥5 times	2.1	20.0	8.7	

**Table 3:** Consumption of processed and ultra-processed food by infants registered in basic family health units. Uberlândia City, Minas Gerais State, 2012.

Food	Age group (months)			p-value
	<6	6 to 11.9	12 to 23.9	
	%			
Cookies				
Not introduced	90.7	33.7	15.0	<0.001
<5 times	8.3	42.5	53.8	
≥5 times	1.0	23.8	31.2	
Starch cookies				
Not introduced	92.8	53.8	58.8	<0.001
<5 times	4.1	31.1	28.8	
≥5 times	3.0	15.0	12.5	
Bread				
Not introduced	97.9	51.3	26.3	<0.001
<5 times	2.1	27.5	16.3	
≥5 times	0.0	21.2	57.5	
Greengrocers				
Not introduced	99.0	90.0	73.8	<0.001
<5 times	1.0	10.0	20.0	
≥5 times	0.0	0.0	6.2	
Cakes				
Not introduced	98.9	85.0	68.8	<0.001
<5 times	0.0	15.0	28.8	
≥5 times	1.0	0.0	2.5	
Cheese bread				
Not introduced	96.9	83.8	68.8	<0.001
<5 times	2.1	13.7	26.3	
≥5 times	1.0	2.5	5.0	
Ice-cream				
Not introduced	100.0	92.5	88.8	0.005
<5 times	0.0	7.50	11.2	
≥5 times	0.0	0.0	0.0	
Salty crackers				
Not introduced	99.0	88.8	57.4	<0.001
<5 times	1.0	11.2	33.8	
≥5 times	0.0	0.0	8.8	
Pasta				
Not introduced	97.9	87.5	70.0	<0.001
<5 times	2.1	12.5	28.8	
≥5 times	0.0	0.0	1.2	
Baby food				
Not introduced	91.8	53.8	41.3	<0.001
<5 times	3.1	11.3	10.0	
≥5 times	5.1	35.0	48.7	

**Table 4:** Consumption of ultra-processed liquids and beverages by infants (G4) registered in basic family health units. Uberlândia City, Minas Gerais State, 2012.

Food	Age group (months)			p-value
	<6	6 to 11.9	12 to 23.9	
	%			
Chocolate powder				
Not introduced	100.0	92.5	61.3	<0.001
<5 times	0.0	5.0	11.2	
≥5 times	0.0	2.5	27.5	
Soft drinks				
Not introduced	100.0	85.0	55.0	<0.001
<5 times	0.0	13.8	41.3	
≥5 times	0.0	1.2	3.7	
Sweetened juice*				
Not introduced	98.0	87.5	91.3	0.049
<5 times	1.0	7.5	2.5	
≥5 times	1.0	5.0	6.2	
Artificial juice**				
Not introduced	96.9	87.5	52.5	<0.001
<5 times	3.1	11.3	36.3	
≥5 times	0.0	1.2	11.3	
Ready milk				
Not introduced	90.7	45.0	23.8	<0.001
<5 times	7.2	38.8	55.0	
≥5 times	2.0	16.2	21.2	
Infant formulas				
Not introduced	61.9	76.3	93.8	0.001
<5 times	3.1	2.5	0.0	
≥5 times	35.0	21.2	6.2	

*nectar juice; **juice powder.

For children aged from 6 to 11.9 months old, the most frequently consumed G1 food were rice (57.5%), beans (73.7%), meat (62.5%), vegetables (86.2%), roots (51.2%), and fruits (80%). Most children had not yet eaten egg (65%), milk (60%) and coffee/tea (63.8%). As for culinary ingredients, oil (76.3%) and salt (85%) were consumed on a regular basis by most children; sugar was regularly consumed by 30% of them. In relation to processed and ultra-processed food for this age group, those that had the highest frequency of regular consumption were cookies (23.8%), bread (21.2%), formulas (21.2%), and baby food (35%). Food, such as greengrocers, cakes, ice cream, chips and pasta were not consumed on a regular basis by any child.

All children aged from 12 to 23.9 months old had already received G1 food, such as rice, meat, vegetables, fruits and pasta. Among these varieties, those that stood out in regular consumption were rice (95%), beans (92.5%), meat (87.5%), vegetables (93.7%), and fruits (91.2%). Oil was consumed regularly by 98.8% of the children; salt, by 100% of them on a regular basis. As for sugar, 48.8% of children consumed it regularly. Among food from G3 and G4, those with the highest regular consumption were cookies (31.2%), bread (57.5%), chocolate powder (27.5%) and baby food (48.7%).

## DISCUSSION

In this study, the lowest median of food offered was six months for *in natura*/minimally processed food (G1). The median of most G4 food was 12 months. After six months old, regular consumption of *in natura* food increased considerably, representing an expressive part of the diet of these children; this consumption is higher than that of processed and ultra-processed food.

The prevalence of EBF found in this study was lower than that found in the Brazilian National Health Survey (PNS), with 24.9% versus 36.6%, respectively, and that of BF was higher (72.3% versus 52.1%, respectively).^18^ In Brazil, there was an upward trend in the prevalence of EBF and BF until 2006, with a possible stability between 2006 and 2013. The median duration also showed a positive trend during this period (2.5 months old in 1975 and 11.3 months old in 2008).^19^

The progress observed in BF are the result of several breastfeeding initiatives carried out in Brazil after 1980.^20^ These advances are one of the factors that helped in the intense changes in the child health profile between the 1970s and 2010s. The drop in infant mortality is explained by a set of health, social and educational policies, especially the FHS, which expanded the access to health care, mainly for the most vulnerable and poor population of the municipalities, and income transfer programs, such as *Bolsa Família*, a Brazilian aid to dependent children, which through its specific conditions linked its beneficiaries to health services.^21^ More recently, as to complementary healthy feeding, the National Strategy for Complementary Healthy Eating (ENPACS) ^22^ and the Brazilian Breastfeeding and Food Strategy stand out^23^. BF and complementary healthy eating are the two feeding practices recommended in the National Food and Nutrition Policy (PNAN) to ensure health and proper development in childhood and adulthood.^24^

In the present study, the median age of salt and oil introduction (seven months old) was found to be similar to that of *in natura* food, since they are used in their preparation. The lowest median of food introduction and the highest frequency of weekly consumption in the two age groups for rice, beans, roots, meat, pasta, vegetables and fruits could be indirectly explained by greater maternal knowledge of complementary feeding and, possibly, by their participation in the orientation groups during childcare consultations, focusing on breastfeeding and healthy eating. In addition, the families of these children belonged to the lowest economic classes, in which the consumption of *in natura* food is lower than in upper classes, according to data from the Family Budget Survey (2008‒2009), and considering that the child’s eating habits probably reflects habits.^25^ The family plays a fundamental role in the construction of their children’s eating habits, and the family nucleus is the first social influence on their diet.^26^ A cohort study conducted in Pelotas City, Rio Grande do Sul State, found that adequate eating practices, such as EBF and adequate introduction of complementary feeding were associated with lower consumption of ultra-processed foods at six years of age.^27^

Processed and ultra-processed food had a median introduction of ten and seven months, respectively, and consumption increased gradually with age. For cookies, introduction occurred before 18 months old for 100% of children. In Brazil, 52% of Brazilian children aged from six to 59 months old consumed bread, a habit that reflects a Brazilian dietary pattern.^9^ In the present study, bread was one of the processed food that had a higher regular consumption among children from six to 23.9 months old.

Gelatin, chocolate, candies, chips, artificial juice, sausages and pasta had a median introduction of 12 months, characterizing an early introduction of this food group, seen that the recommendation is after 24 months old. ^22^ The median for sugar was seven months old, indicating that, in addition to the sugar present in processed and ultra-processed food, there is possibly added sugar in milk, juice and other food prepared by the family.

Results similar to those found in this study were verified in a study conducted in Acrelândia City, Acre State, with children under two, cared for at the FHS, with an increasing consumption of food not recommended for their age group.^28^ Another study conducted in 48 municipalities participating in the Brazil Without Misery Plan, in Southern Brazil, showed that 35.5% of children ate sugar before they were 4 months old, and that the prevalence of introducing sweet/salty crackers, petit swiss cheese and gelatin before they were six months old was 20.4%; 24.8% and 13.8%, respectively.^29^ In the present study, most children under six months old did not eat sugar or processed and ultra-processed food.

The main limitations of this study are listed in this paragraph. The first is cross-sectional, since longitudinal studies would be more appropriate to collect the age of food introduction, because introducing food occurs simultaneously with the report, reducing the recall bias. We believe that the possibility of recall bias may have been higher for children who had already been exposed to food when this introduction occurred at an early age and together with other types of food. However, maternal memory for reporting infant feeding is a valid and reliable estimate over a period of up to three years.^30^ Although few children attend school (6.7%), it was not possible to identify if the mother’s report was reliable for those who stay full time (5.7%). Possibly, the age of introduction reported by the mother may have been overestimated, considering it is more common for children to have already eaten some food unknown to their mothers. The frequency of regular consumption (≥5 days/week) may have been underestimated, because mothers base their information on the observation of consumption at home, which can be lower than at school.

Another highlight is the influence of the percentage of children who were not exposed to some types of food (considered in this study as a case of censorship) on the median overestimation. However, this analysis is adequate for data with incomplete information (when the food has not yet been introduced). Further studies with proposed adjustments should be developed.

A final limitation was the exclusion of children who are cared for in rural units, which could have different characteristics from those served in the urban area. We believe that this limitation was minimized, because there are only two units in the rural area and the population served in all health units have homogeneous characteristics regarding education and economic classification.

In this study, food introduced for the first time and those most frequently consumed were mainly *in natura*, which is in line with recommendations from national and international bodies. The introduction of most processed (G3) and ultra-processed (G4) food occurs early, and they should be offered after 24 months old. We highlight the need for health promotion actions during childcare consultations by trained health professionals to explain the importance of healthy eating practices since early childhood.
